# Prevalence and characteristics of vascular cognitive impairment in a European cohort of adult patients with Moyamoya angiopathy

**DOI:** 10.1007/s00415-024-12555-2

**Published:** 2024-07-17

**Authors:** Marine Giroud, Lionel Calviere, Carla Machado, Sonia Reyes, Hélène Mirabel, Nicolas Raposo, Pierre Brandicourt, Alain Viguier, Jean-François Albucher, Fabrice Bonneville, Jean Marc Olivot, Patrice Péran, Jérémie Pariente, Dominique Hervé, Mélanie Planton

**Affiliations:** 1 Neurology Department, Toulouse University Hospital, Toulouse, France; 2 Toulouse NeuroImaging Center ToNIC, Toulouse University, Toulouse, France; 3 Neurology Department, Hospital Paris Lariboisière, Paris, France

**Keywords:** Moyamoya, Stroke, Vascular cognitive disorders, Neuropsychology

## Abstract

**Introduction:**

Moyamoya angiopathy (MMA) is associated with a high risk of stroke, but it is also increasingly recognized as leading to cognitive impairment. The aim of this study was to determine the prevalence, nature, and severity of vascular cognitive impairment no dementia (VCIND) in adults with MMA and to identify clinical and imaging factors associated with VCIND.

**Methods:**

We conducted a retrospective study of consecutive adult patients with MMA followed in two tertiary hospitals (Toulouse and Paris Lariboisiere). All patients underwent neuropsychological assessment and brain magnetic resonance imaging (MRI). VCIND was defined as at least two variables of the same cognitive process with z-scores of < 2 standard deviations, regardless of the cognitive domain, that do not interfere in everyday life. Baseline demographic, clinical, and imaging data were compared between patients with and without VCIND.

**Results:**

A total of 102 patients (mean age 43 years; 65% women) were included. Thirty-four patients (33.3%) had VCIND. VCIND was mild in 20/34 (59%), moderate in 8/34 (23%), and severe in 6/34 (18%) patients. Executive function was the most widely affected (25.5%), followed by attention and processing speed (24.8%). In univariable analyses, VCIND was associated with ischemic stroke at diagnosis and the presence of ischemic lesions on MRI.

**Conclusions:**

VCIND is highly prevalent in adults with MMA. Executive functions and processing speed are predominantly affected. These findings may guide clinicians in their evaluation of patients with MMA. Further research should assess the effect of revascularization therapies on cognitive functions.

**Supplementary Information:**

The online version contains supplementary material available at 10.1007/s00415-024-12555-2.

## Introduction

Moyamoya angiopathy (MMA) is a rare cerebrovascular pathology characterized by a progressive narrowing and occlusion of the terminal intracranial carotid artery and its main branches, with the development of a basal collateral network [1]. Ischemic and hemorrhagic strokes are the main complication of the disease [2], but other clinical manifestations such as cognitive impairment might distinctly affect patients’ quality of life [3, 4].

A high prevalence of vascular cognitive impairment (VCI), estimated to affect 31% of MMA patients, has been reported in a recent meta-analysis conducted by Kronenburg et al. [4] But, according to the latest data provided by Chan et al., it may be even more common: their findings report that 75% of patients have VCI [5]. Cognitive data remain, however, limited notably because of the small sample size, and interpretation is still complicated in the absence of an identified consensual neuropsychological pattern and a quantitative definition of VCI. Few studies have suggested a preferential impairment of executive function, especially mental flexibility function [3, 5–11], followed by processing speed [3, 5, 7]. Data on memory are even more scarce and feature conflicting results: some studies have reported normal functioning [3] while others have found that memory impairment affects one third of patients [5, 7]. Moreover, the mechanisms underlying cognitive impairment remain controversial. In addition to the impact of ischemic or hemorrhagic lesions on cognitive functioning [12, 13], chronic cerebral hypoperfusion is thought to play a role. Some preliminary results suggested an association between perfusion and cognition in patients with MMA [6, 14–16].

In this study, we aimed to determine the frequency, nature, and severity of vascular cognitive impairment no dementia (VCIND) in a large bi-centric cohort of adult patients with MMA. We also evaluated the clinical and imaging factors associated with cognitive impairment in patients MMA.

## Methods

We conducted a retrospective, bi-centric study of consecutive adult patients with MMA followed in two tertiary hospitals (Paris Lariboisiere University Hospital and Toulouse University Hospital). The study periods extended from March 2008 to July 2021 in Toulouse and January 2015 to December 2020 in Paris.

### Standard protocol approvals, registrations, and patient consents

According to French ethics and regulatory law, retrospective studies based on the exploration of usual care data should not be submitted to an ethics committee but they must be declared or covered by the reference methodology of the French National Commission for Informatics and Liberties (CNIL). Patients were informed that their codified data would be used for the study. After evaluation and validation by the data protection officer and according to the General Data Protection Regulation, this study met all the criteria. It is registered in the retrospective studies registry of Toulouse University Hospital (registration number: RnIPH 2021-65) and covered by the MR-004 (CNIL number: 2121529 v1).

### Participants

Patients were eligible for the study if they fulfilled the following criteria: ‘(1) they were adults (over 18 years of age) diagnosed with MMA disease or syndrome according to the established criteria of the Research Committee on the Pathology and Treatment of Spontaneous Occlusion of the Circle of Willis [17]; (2) they had an available neuropsychological assessment (NPA) prior to any revascularization surgery and at least 3 months after the onset of stroke in patients with stroke presentation; (3) they had an available brain MRI of adequate quality performed within 6 months of NPA.

Patients were excluded from our analyses if they had any comorbidities that could be responsible for cognitive impairment and/or if their visual, auditory, and verbal or written language skills were insufficient for performing the neuropsychological tests correctly. Patients with major vascular cognitive impairment (i.e., dementia) and/or total dependence according to the DSM-5 criteria were not eligible for this study due to the lack of available neuropsychological data for an exhaustive description.

### Baseline data collection

Baseline demographic, clinical, and radiological characteristics were recorded for all patients. Demographic and clinical characteristics were age at the first NPA, age at the first symptom and diagnosis, delay between first symptom and diagnosis, and Moyamoya disease or syndrome. Vascular risks factors including diabetes, active smoking, high blood pressure (define by the presence of an antihypertensive medication), and hypercholesterolemia (presence of statin treatment or LDL threshold above the target for secondary prevention) were collected. Clinical manifestations leading to the diagnosis of MMA were also recorded: presence of ischemic stroke; transient ischemic attack (TIA); hemorrhagic stroke (subarachnoid hemorrhage, intracerebral hemorrhage); headache; epilepsy; and any other incident findings. It is worth noting that we grouped TIA and ischemic stroke as ischemic presentation and stroke (ischemic or hemorrhagic) plus TIA as vascular manifestations. Finally, radiological features on MRI (DWI, FLAIR, T2*, TOF sequences) were independently assessed for the absence or presence of uni- or bilateral vascular impairment, ischemic or hemorrhagic lesions on MRI, unilateral or bilateral hemispheric lesions on MRI, and the involvement of posterior circulation. The presence of transdural collaterals was identified by digital subtraction angiography, if performed.

### Neuropsychological assessment

All patients underwent a comprehensive neuropsychological assessment. The assessments were performed by experienced neuropsychologists. The neuropsychological test battery was standardized across the participating study centers for patients evaluated after 2016. All tests and assessment techniques are detailed in Lezak [18]. Table 1 shows the mean cognitive z-scores and the percentage of patients who completed each test (with min–max bounds of 56 to 98%). Cognitive tests were grouped by cognitive domains (executive functions, memory, attention and processing speed). Executive domain included the initiation function explored by the verbal fluency test (animals and letter P), mental flexibility was tested by the Trail Making Test, inhibition by the Stroop test, and verbal and visuo-spatial working memory was tested using the digit spans subtests of the Wechsler scales (WAIS-IV). The D2 task was performed to assess selective attention. Processing speed was explored with the subtest Code of the WAIS-IV, the time of the TMTA and the times scores at the denomination and reading conditions of the Stroop test. Finally, and because of reduced acquisition of anterograde memory scores, we grouped visual and verbal data according to the nature of the process involved but independently of the stimuli modality. In this way, we used the immediate recall score of the Free and Cued Selective Reminding Test (FCSRT) for the encoding process, while we used the free delayed recall of the FCSRT and the total recall score of the BVMT-R test for retrieval process. The storage process was explored using the total delayed scores of the FCSRT and BVMT-R tests.

**Table 1:** Mean cognitive z-scores and percentage of patients completing each test. *Normative data reference average performance to a percentile above 50

	Cognitive variable	% available data	Mean z-score
**Executive**			
Initiation	Verbal fluency (animals)	93	− 0.58
	Verbal fluency (P)	93	− 0.59
Flexibility	TMT B time (sec)	96	− 1.10
	TMT B-A time	97	− 0.77
	TMT B-A errors	85	− 0.71
Inhibition	Stroop test interference score (IS), time	87	− 1.24
	Stroop test interference-denomination, time	84	− 0.93
	Stroop test IS, non-corrected errors	65	− 0.45
Working memory	Digit span forward	97	− 0.50
	Digit span backward	97	− 0.51
	Visual span forward	56	− 1.51
	Visual span backward	56	− 1.26
**Attention/processing speed**			
Selective attention	D2 GZ	59	− 0.80
	D2 F%	59	0.20
	D2 Gz-F	59	− 0.77
Processing speed	WAIS code	69	− 0.60
	TMT A time (sec)	98	− 0.46
	Stroop denomination time (sec)	90	− 1.17
	Stroop reading time (sec)	89	− 1.54
**Anterograde memory**			
Encoding/retrieval processes	FCRST immediate recall (/16)	97	0.0*
	FCRST free delayed recall (/16)	97	− 0.57
	BVMTR total recall (/36)	65	0.66
Storage process	BVMTR delayed recall (/12)	64	0.28
	FCRST total delayed recall (/16)	97	− 0.67

#### Definition and severity of vascular cognitive impairment no dementia

VCIND was defined as impairment in at least one cognitive function with at least two z-scores strictly below 2 standard deviations (SD) according to the clinical norms, whatever the cognitive domain investigated without interference in the activities of daily life.

The severity of cognitive impairment was determined by calculating the percentage of pathological variables that were individually weighted based on the total number of cognitive variables assessed. Specifically, VCIND was subclassified as mild if less than 30% of the cognitive scores were altered, moderate if between 30 and 50% of the scores were altered, and severe if more than 50% of the scores were altered.

### Statistical analysis

Demographic, clinical, and neuropsychological data were described as means and standard deviations (SD) or medians and interquartile ranges (IQR) for quantitative variables and as numbers and percentages for qualitative variables, when appropriate.

As detailed, the prevalence of VCIND was calculated as the proportion of patients who fulfilled the definition. To explore the impact of strokes on cognition, considering our hypothesis that stroke events negatively affect cognitive performance, we plotted cognitive z-scores of patients with Moyamoya angiopathy, stratified by the presence or absence of a history of stroke. In addition, to investigate which demographic, clinical, and radiological factors might play a significant role in determining the presence or absence of VCIND in patients with MMA, we performed 2-sided tests with the presence of significant cognitive impairment as the dependent variable. The same comparisons were then repeated for each cognitive domain investigated (presence or absence of executive/attention-speed domains), except for the memory domain, as the distribution of patients between the groups did not meet the assumptions for statistical comparison. For the latter analyses, we used the χ^2^ test for categorical variables and the Mann–Whitney U test for continuous variables, as appropriate.

Results were considered statistically significant at an α level of 0.05. All statistical analyses were performed with SPSS 14.0 software (IBM corporation, 2016).

## Results

### Population characteristics

A total of 102 patients were included during the study period. Forty-four were included from Paris University Hospital and 58 from Toulouse University Hospital.

Sixty-six patients were women (65%) and the majority were Caucasian (71%) (Table 2). The mean age at diagnosis and at neuropsychological assessment were 39.4 (± 18.5) and 43 (± 13) years, respectively. Sixty-four patients had Moyamoya disease (63%) and 38 (37%) had Moyamoya syndrome (atherosclerosis (n = 9); vasculitis (n = 5); neurofibromatosis type 1 (n = 2); sickle cell disease (n = 2); beta thalassemia (n = 2); other (n = 18)). The arterial lesions were considered as bilateral in 63 patients (62%).

**Table 2:** Clinical and radiological characteristics of patients with MMA

Clinical characteristics	
Number of patients	102
Paris hospital	44
Toulouse hospital	58
Men, n (%)	36 (35)
Age at first symptom, mean (SD)	35.9 ± 23.8
Age at diagnosis, mean (SD)	39.4 ± 18.5
Age at first neuropsychological assessment, mean (SD)	43 ± 13
Years of study, median (IQ)	11 (11–14)
Delay first symptom/diagnosis (day), median (IQ)	68 (0–497)
Ethnicity, n (%)	
Caucasian	72 (70)
North African	12 (12)
Asian	10 (10)
Other	8 (8)
Moyamoya angiopathy subtype, n (%)	
MMA disease	64 (63)
MMA syndrome	38 (37)
Bilateral MMA	63 (62)
Family history, n (%)	2 (2)
Clinical presentation, n (%)	
All strokes	63 (62)
Ischemic stroke	48 (47)
Hemorrhagic stroke	15 (14)
TIA	11(10)
Headache	10 (9)
Incidental	17 (16)
Epilepsy	1 (1)
Vascular risk factors, n (%)	
Hypertension	30 (29)
Smoking	37 (36)
Dyslipidemia	22 (22)
Imaging characteristics, n (%)	
Presence of transdural anastomosis	21 (21)
Vertebro-basilar involvement	16 (16)
Presence of one or more ischemic lesions	75 (74)
Presence of one or more hemorrhagic lesions	16 (16)

Clinical presentations at diagnosis included stroke in 63 out of the 102 (62%) patients (n = 48 (47%) ischemic stroke, n = 15 (15%) hemorrhagic stroke) and non-stroke presentation in 38 out of the 102 (38%) patients.

### Prevalence and pattern of vascular cognitive impairment no dementia

Thirty-four patients out of the 102 patients (33.3%) were diagnosed as having VCIND. Executive domain was most commonly impaired (n = 26 (25.5%)) followed by attention and processing speed (n = 25 (24.8%)). Based on cognitive function, the most impaired functions were processing speed (21.2%), followed by inhibition (16.3%), and mental flexibility (16.3%). Alterations in other cognitive functions were 10.1% for selective attention, 3.2% for initiation, 1.5% for storage memory, and 1% for working memory and encoding/retrieval memory processes. Details of mean z-scores are given in Table 1.

### Severity of vascular cognitive impairment no dementia

According to our criteria, VCIND was classified as mild in 20 out of 34 patients (59%), moderate in 8 patients (23%), and severe in 6 patients (18%). Among patients with VCIND, the most impaired cognitive functions were processing speed (53%), inhibition (33%), and flexibility (30%). In patients with moderate-to-severe impairment, as in mild impairment, the most impaired cognitive functions were processing speed (85%), flexibility (83%), and inhibition (82%).

### Determinant of vascular cognitive impairment no dementia

Stroke at diagnosis (χ^2^ = 9.15, *p* = 0.002), including ischemic stroke (χ^2^ = 6.38, *p* = 0.012) and ischemic lesions on MRI at baseline (χ^2^ = 5.67, *p* = 0.017) were associated with VCIND (Table 3).

**Table 3:** Univariate analysis of patients with MMA with cognitive impairment

	No VCIND	VCIND	*P* value
Number of patients	68	34	
**Clinical characteristics**			
Men, n (%)	25 (37)	11 (32)	0.66
Age at diagnosis, mean (SD)	39.4 ± 20.4	39.5 ± 14	0.92
Age at first neuropsychological assessment, mean (SD)	42.8 ± 13.5	43.3 ± 12.2	0.77
Delay first symptom/diagnosis (day), median (IQ)	45 (1–653)	85.5 (0–431)	0.79
MMA disease, n (%)	41 (60)	23 (68)	0.47
Vascular event at diagnosis, n (%)	45 (66)	29 (85)	0.041*
TIA and ischemic strokes	36 (53)	23 (68)	0.57
Ischemic stroke	26 (38)	22 (65)	0.012*
All strokes	35 (51)	28 (82)	0.002*
Hypertension, n (%)	20 (29)	10 (29)	1.00
Smoking, n (%)	24 (35)	13 (38)	0.77
Dyslipidemia, n (%)	12 (18)	10 (29)	0.19
**Radiological characteristics**			
Bilateral involvement, n (%)	41 (60)	22 (65)	0.67
Transdural involvement, n (%)	15 (22)	6 (18)	0.60
Vertebro-basilar involvement, n (%)	8 (12)	8 (24)	0.12
Presence of ischemic lesions on MRI, n (%)	45 (66)	30 (88)	0.017*
Presence of hemorrhagic lesions on MRI, n (%)	10 (15)	6 (18)	0.70

When factors were analyzed according to a specific cognitive domain of interest, we observed that symptomatic stroke at diagnosis (*p* = 0.007) including ischemic stroke (*p* = 0.018) as clinical features were associated with impairment of the attention and processing speed domain. Impairment in the executive domain was related to the presence of chronic ischemic lesions on MRI at baseline (*p* = 0.046). For details, see supplementary tables S1 and S2.

**Fig. 1:**
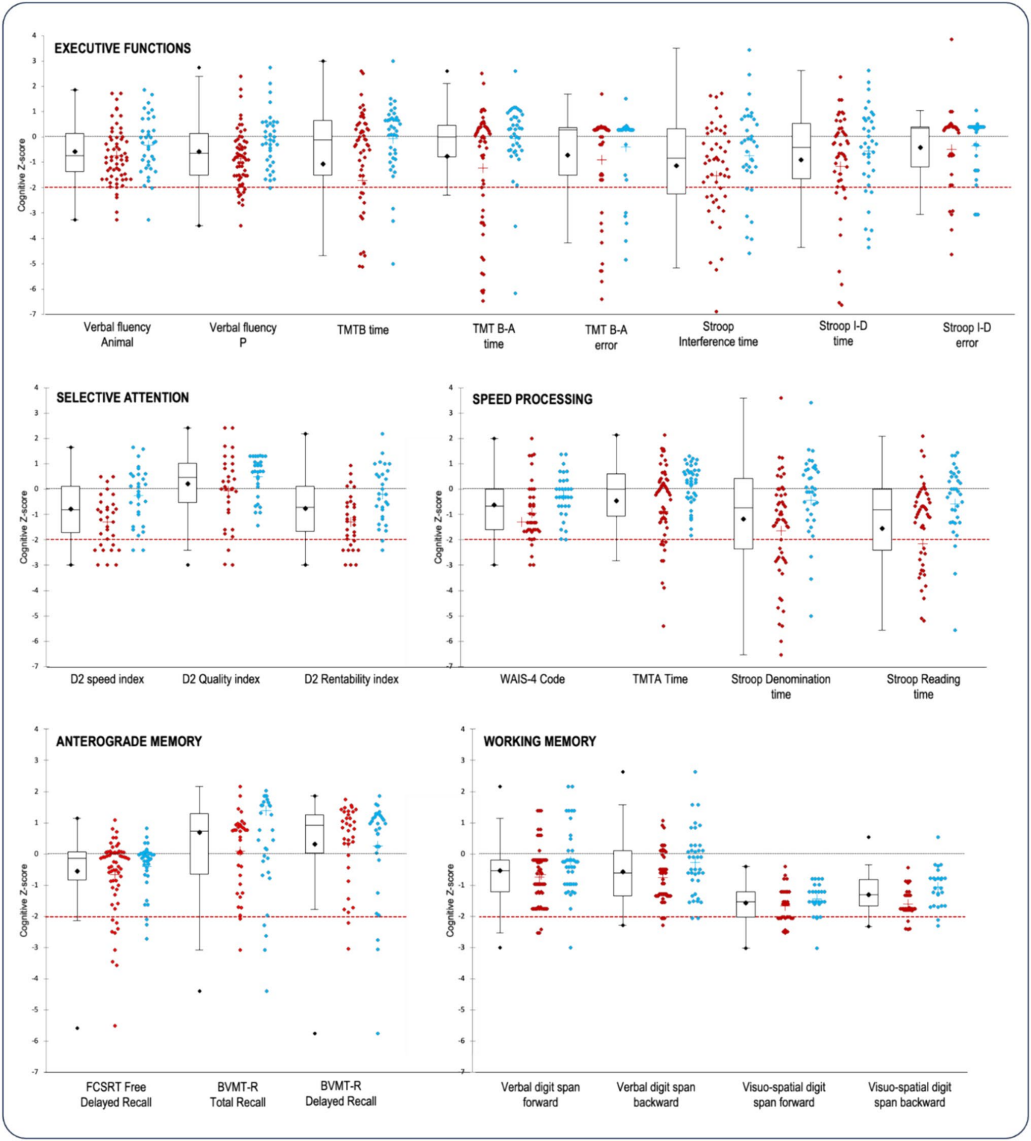
Distribution of cognitive z-scores taking into account the presence of stroke at diagnosis (MMA patients with stroke in red, without stroke in blue). The dotted line represents the pathological threshold of –2SD. The dot plot represents the mean, the median, the first and third interquartile range, and the standard error of the whole population. The cross represents the mean of each group. None of the cognitive processes examined fell below the threshold of -2SD. When considering the group as a whole, the cognitive domains of speed, inhibition, and working memory exhibited the lowest mean z-scores, suggesting potential areas of impairment. The distributions of scores on the cognitive functions of speed, inhibition, mental flexibility, and anterograde memory were highly variable in MMA patients, indicating a wide range of performance levels within these areas. Interestingly, the presence of a stroke did not lead to more severe cognitive impairment at the group level. This highlights the influence of other factors on cognitive impairment and emphasizes the importance of considering a range of factors beyond stroke history when understanding cognitive outcomes in patients with MMA

In addition, we observed a similar distribution of cognitive scores between MMA patients with and without a medical history of symptomatic stroke (Fig. 1).

## Discussion

To our knowledge, this study includes the largest cohort to date of adult patients with MMA explored with detailed neuropsychological assessment. Our findings further support a high prevalence of vascular cognitive impairment no dementia, affecting approximately one third of adult patients with MMA. In addition, our results provide additional insights into the neuropsychological profile of MMA, demonstrating a predominant impairment in mental flexibility, inhibition, and processing speed functions.

In this large cohort of adult patients with MMA, we found that about 33% of them experienced VCIND. This finding aligns with the results of previous investigations. For instance, in a smaller study of 36 patients, Karzmark et al. reported a similar rate of cognitive impairment of 31% [3]. Furthermore, a recent meta-analysis conducted by Kronenburg et al. also estimated an average rate of cognitive impairment of 31%. In this latter study, however, there was substantial variation between the five included studies, with rates ranging from 0 to 69% [3, 6, 7, 19, 20]. These rates are considerably lower than the results reported by Chan et al., which suggests a much higher prevalence of cognitive impairment (75%) [5]. Several factors may account for the divergent VCI rates observed between trials. Variations in patient selection, including factors such as the presence or absence of stroke, the specific type of stroke, or patients with previous surgical revascularization, may contribute to these discrepancies. Additionally, differences in the definition of VCIND used by the authors may also influence the reported rates. When we used a less restrictive definition of VCIND, in which only one pathological variable was required, we found a rate of 70%. However, we are aware that in this study we underestimated VCI by selecting only a proportion of the MMA population, in particular those eligible for neuropsychological assessment. The inclusion of patients with severe aphasia and/or patients with dementia would have increased our prevalence. The retrospective design of this study does not allow us to analyze these data in detail, but our findings certainly call for replication.

One of the major challenges in cognitive studies of MMA is the lack of a consensual definition of cognitive impairment. While the definition of VCIND can be applied, there is still a lack of clarity regarding the specific quantitative criteria for impairment in each cognitive function and/or domain. In our study, we aimed to propose a definition that was sensitive enough to detect VCIND but not overly permissive. Considering the potential impact of fatigue resulting from the long duration of neuropsychological assessment and the possibility of impairment after stroke, we chose a threshold of -2 standard deviations, which is more stringent than many studies in this field. We also based our criteria on the presence of at least two variables below this threshold to define impairment, drawing from previous studies conducted by our group in VCI [21], which demonstrated robust external validity. Notably, when we apply the criteria for cognitive impairment used by Karzmark et al. (which require at least 50% of variables with DS ≤1 DS), we observe a similar rate of VCIND of 31%, suggesting that our definition may be appropriate and reproducible. These findings are also consistent with the reported prevalence of cognitive impairment in young adults who have experienced strokes, which is approximately 35%, as reported by Schaapsmeerders et al. [22].

Several studies have found a strong relationship between frontal areas and executive function [23–32], supporting the hypothesis that executive impairment is predominant in MMA. The Trail Making Test Part B (TMT-B), which assesses mental flexibility, has been consistently impaired in MMA studies [3, 6, 7], as has processing speed in approximately 30% of patients. Our results confirmed this pattern of executive dysfunction, particularly in flexibility, inhibition, and processing speed functions. This is consistent with the known localization of lesions and hypoperfusion in MMA, with frontal areas being particularly affected by stenosis in the anterior vascular network. In our study, we observed that memory functioning seemed to be spared. Due to methodological constraints and given the involvement of the frontal lobes in the quality of encoding and their contribution to retrieval mechanisms, we chose to group our memory scores according to the processes involved rather than the modality of item presentation (e.g., verbal vs non-verbal). Despite this classification, we found no evidence of memory impairment in adults with MMA. These findings are consistent with previous studies conducted by Karzmark et al. in 2008 and 2012. However, they contradict the findings reported by Festa et al. in 2010 and Kazumata et al. in 2015, where an altered memory process was observed in approximately one third of the patients. It is worth noting that the results of the latter study are in line with Kronenburg's recent research, which also found a high rate of impairment in 37% of patients. Despite this rate, the mean standard deviation values at group level were estimated to be within the strict normal range. Given these discrepancies, it is important to emphasize that this research needs to continue to be replicated [33, 34].

The relationship between cognitive impairment and stroke has been well documented in the literature [12, 35], particularly in the context of strategic lesions [32, 36] and extensive white matter damage [32]. However, few studies have specifically investigated this relationship in MMA [4]. Although our qualitative analysis, focusing on the distribution of z-score values, did not reveal clear differences between patients with and patients without stroke, it is essential to acknowledge that strokes are not the only factor contributing to cognitive impairment in MMA. Other factors, such as the burden of asymptomatic lesions observed through MRI and the involvement of chronic cerebral hypoperfusion, are also believed to play a significant role [6, 10, 14, 37–39]. The degree of hypoperfusion in the frontal area may account for some of the impairments in executive functions and processing speed by disrupting cerebral networks through the presence of microstructural white matter lesions [6, 19, 40–42].

There are several limitations to this study. Firstly, because of its retrospective design, our prevalence may be misestimated, although given that our results are in agreement with the literature, our estimate may not be too far off at all. Secondly, the NPA protocols were not standardized between the two participating centers until 2016. Despite this, we attempted to control for heterogeneity by using z-scores and grouping variables by cognitive function. Thirdly, there was often a significant delay (median 262 days [113–709]) between diagnosis and the first NPA, which may have potentially influenced the results. Patients were typically diagnosed in a stroke unit and subsequently referred to our reference center for NPA, resulting in varying time intervals. Finally, in a clinical setting, imaging protocols were not standardized across study centers. We were, therefore, unable to evaluate the effects of MRI brain lesions and hypoperfusion on cognitive functions.

## Conclusion

Our study, conducted in a large cohort, confirms the high prevalence of cognitive impairment in adults with MMA, estimated to affect around one third of all patients. We have identified a specific pattern of cognitive deficits that predominantly impact executive functions and processing speed. Stroke and MRI lesions are significant determinants of cognitive impairment in MMA, consistent with the pathophysiology of the disease and the primary involvement of frontal areas. Harmonizing definitions and test batteries in MMA is essential in improving both how young patients can be cared for and how their daily life can be adapted in the light of their condition. Further research is needed in order to explore the underlying mechanisms, the effect of hypoperfusion, and the impact of revascularization surgery on cognitive impairment in MMA.

## Supplementary Information

Below is the link to the electronic supplementary material.

Supplementary Material 1.

## Data Availability

Data are available upon request.
