# Supplementary material for: Prevalence and characteristics of vascular cognitive impairment in a European cohort of adult patients with Moyamoya angiopathy
Source: J Neurol. 2024 Jul 17;271(9):5976–84. doi: 10.1007/s00415-024-12555-2 (PMC11377615; doi:10.1007/s00415-024-12555-2)
Supplement: Supplementary file 1 — Supplementary Material 1. [file 415_2024_12555_MOESM1_ESM.pdf]

Prevalence and characteristics of vascular cognitive impairment in a European cohort of adult patients with Moyamoya angiopathy

Journal of Neurology

Marine Giroud (MD) [1]; Lionel Calviere (MD) [1,2]; Carla Machado (MSc) [3]; Sonia Reyes (MSc) [3]; Hélène Mirabel (MSc) [1]; Nicolas Raposo (MD, PHD) [1,2]; Pierre Brandicourt (MD) [4]; Alain Viguier (MD) [1,2]; Jean-François Albucher (MD) [1,2]; Fabrice Bonneville (MD, PHD) [2,5]; Jean Marc Olivot (MD, PHD) [1,2]; Patrice Péran (PHD) [2] ; Jérémie Pariente (MD, PHD) [1,2]; Dominique Hervé (MD) [3]; Mélanie Planton (PHD) [1,2]

1. Neurology Department, Toulouse University Hospital, France

2. ToNIC, Toulouse NeuroImaging Center, Toulouse University, France

3. Neurology Department, Hospital Paris Lariboisière, France

4. Neuroradiology Department, Toulouse University Hospital, France

5. Neuroradiology Department, Toulouse University Hospital, France

Marine GIROUD, Neurology Department, Toulouse University Hospital, France; giroud.m@chu-toulouse.fr

	Executive domain		
	No VCIND	*VCIND*	*P-value*
Number of patients	76	26	
**Clinical characteristics**			
Men, n (%)	27 (36)	9 (35)	0.93
Age at diagnosis, mean (SD)	39.2 ± 19.8	40.1 ± 14.2	1.00
Age at first neuropsychological assessment, mean (SD)	43 ± 13.4	42.8 ± 12	1.00
Delay first symptom/diagnosis (day), median (IQ)	68 (1-656)	49 (0-190.8)	0.31
Disease, n (%)	46 (61)	17 (65)	0.66
Vascular event at diagnosis, n (%)	53 (70)	21 (81)	0.28
TIA and ischemic strokes	43 (57)	16 (62)	0.66
Ischemic stroke	33 (43)	15 (58)	0.20
All strokes	43 (57)	20 (77)	0.07
Hypertension, n (%)	24 (32)	6 (23)	0.41
Smoking, n (%)	28 (37)	9 (35)	0.84
Dyslipidemia, n (%)	15 (20)	7 (27)	0.46
**Radiological characteristics**			
Bilateral involvement, n (%)	47 (62)	16 (62)	0.98
Transdural involvement, n (%)	17 (22)	4 (15)	-
Vertebro-basilar involvement, n (%)	10 (13)	6 (23)	0.23
Presence of ischemic lesions on MRI, n (%)	52 (68)	23 (88)	0.046*
Presence of hemorrhagic lesions on MRI, n (%)	11 (14)	5 (19)	0.57

Table S1: Clinical and radiological characteristics of patients with MMA with and without executive impairment

	Speed processing/attention domain		
	No VCIND	*VCIND*	*P-value*
Number of patients	76	25	
**Clinical characteristics**			
Men, n (%)	29 (38)	6 (24)	0.20
Age at diagnosis, mean (SD)	38.8 ± 19.6	40.7 ± 14.9	0.46
Age at first neuropsychological assessment, mean (SD)	42.1 ± 13.2	45 ± 12.5	0.21
Delay first symptom/diagnosis (day), median (IQ)	45 (1-497)	133 (0-666.5)	0.88
Disease, n (%)	47 (62)	16 (64)	0.85
Vascular event at diagnosis, n (%)	51 (67)	22 (88)	0.043*
TIA and ischemic strokes	41 (54)	18 (72)	0.11
Ischemic stroke	31 (41)	17 (68)	0.018*
All strokes	41 (54)	21 (84)	0.007*
Hypertension, n (%)	21 (28)	9 (36)	0.43
Smoking, n (%)	25 (33)	12 (48)	0.17
Dyslipidemia, n (%)	13 (17)	9 (36)	0.051
**Radiological characteristics**			
Bilateral involvement, n (%)	47 (62)	16 (64)	0.85
Transdural involvement, n (%)	16 (21)	5 (20)	0.94
Vertebro-basilar involvement, n (%)	10 (13)	6 (24)	0.20
Presence of ischemic lesions on MRI, n (%)	53 (70)	22 (88)	0.07
Presence of hemorrhagic lesions on MRI, n (%)	11 (14)	4 (16)	-

Table S2: Clinical and radiological characteristics of patients with MMA with and without attention/speed impairment
